# Supplementary material for: Content-rich biological network constructed by mining PubMed abstracts
Source: BMC Bioinformatics. 2004 Oct 8;5:147. doi: 10.1186/1471-2105-5-147 (PMC528731; doi:10.1186/1471-2105-5-147)
Supplement: Additional File 2 — The original results of the above study (non-essential files are deleted to keep the file size under the limit set by BMC bioinformatics). [file 1471-2105-5-147-S2.bz2 › chilibotAdditionalFile2/dip20/275ID1922387E1095/html/MAPK10_JUN.html]

 


 **MAPK10** and **JUN** 
  
Found 55 abstracts in PubMed, retrieved 20.  
 

 What does Google say? 
 PDF only 
| .edu only 

---

**Interactive relationship** (e.g. stimulation, inhibition, etc)

**Stimulatory relationship**- Herein, we provide cDNA microarray evidence that METH administration caused the induction of c  **Jun**  and of other members involved in the pathway leading to c  **Jun**  activation stress activated protein kinase  **Jun**  N terminal kinase JNK3  [ **MAPK10** ] , Crk associated substrate Cas and c Src after environmental stresses or cytokine stimulation.  Ref: 11961130 Mol Pharmacol, 2002
- Here, we show that cdk5 directly phosphorylates c  **Jun**  N terminal kinase 3  [ **MAPK10** ]  JNK3  [ **MAPK10** ]  on Thr131 and inhibits its kinase activity, leading to reduced c  **Jun**  phosphorylation.  Ref: 11823425 EMBO J, 2002
**Neutral relationship**- JNK3  [ **MAPK10** ]  contributes to c  **jun**  induction and apoptosis in 4 hydroxynonenal treated sympathetic neurons.  Ref: 12424734 J Neurosci Res, 2002
- c  **Jun**  N terminal kinase 3  [ **MAPK10** ]  JNK3  [ **MAPK10** ]  stress activated protein kinase beta SAPKbeta binds and phosphorylates the neuronal microtubule regulator SCG10.  Ref: 11718727 FEBS Lett, 2001
- Particularly, JNK3  [ **MAPK10** ]  is considered to be a degenerative enzyme with c  **Jun**  as a relevant substrate.  Ref: 12401814 J Biol Chem, 2003
- Second, we show that HNE induced c  **Jun**  phosphorylation and c  **jun**  induction are attenuated in JNK3  [ **MAPK10** ]  deficient neurons.  Ref: 12424734 J Neurosci Res, 2002

**Non-interactive relationship** (e.g. studied together, co-existance, homology, etc.)

- Although previous evidences indicate that JNK3  [ **MAPK10** ]  is required for neuronal death under certain conditions, we show that JNK3  [ **MAPK10** ]  deficiency only partially mediates c  **Jun**  phosphorylation and its deficiency does not protect neurons from death.  Ref: 12091388 J Biol Chem, 2002
- When characterizing the 5 flanking region of the c  **Jun**  NH2 terminal kinase 3 gene at 4q21 22, where frequent allelic losses and loss of expression had been detected in patients with brain tumors and hepatocellular carcinomas, we discovered that the Fas associated phosphatase 1 gene was located only 633 bp upstream from JNK3  [ **MAPK10** ]  in a head to head orientation.  Ref: 12436199 J Hum Genet, 2002
- In summary, these results indicate that JNK3  [ **MAPK10** ]  plays a critical role in HNE induced c  **Jun**  activation and apoptosis in sympathetic neurons.  Ref: 12424734 J Neurosci Res, 2002
- Here, we report that p75 activates Rac GTPase, which in turn activates c  **jun**  N terminal kinase JNK, including an injury specific JNK3  [ **MAPK10** ] , in an NGF dependent manner.  Ref: 11756498 J Neurosci, 2002
- Moreover, cdk5 deficient cultured cortical neurons exhibit increased sensitivity to apoptotic stimuli, as well as elevated JNK3  [ **MAPK10** ]  activity and c  **Jun**  phosphorylation.  Ref: 11823425 EMBO J, 2002
- Cyclin dependent kinase 5 prevents neuronal apoptosis by negative regulation of c  **Jun**  N terminal kinase 3  [ **MAPK10** ] .  Ref: 11823425 EMBO J, 2002
- c  **Jun**  N terminal kinase 3  [ **MAPK10** ]  JNK3  [ **MAPK10** ] , the only neural specific isoform, may play an important role in excitotoxicity and neuronal injury.  Ref: 12767482 Neurosci Res, 2003
- A single c  **Jun**  N terminal kinase isoform JNK3  [ **MAPK10** ]  p54 is an effector in both neuronal differentiation and cell death.  Ref: 12401814 J Biol Chem, 2003
